# Complementary Techniques of Thermal Analysis as a Tool for Studying the Properties and Effectiveness of Intumescent Coatings Deposited on Wood

**DOI:** 10.3390/polym18020202

**Published:** 2026-01-12

**Authors:** Nataša Čelan Korošin, Romana Cerc Korošec

**Affiliations:** Faculty of Chemistry and Chemical Technology, University of Ljubljana, Večna pot 113, 1000 Ljubljana, Slovenia; natasa.celan@fkkt.uni-lj.si

**Keywords:** thermal stability, flammability, flame retardancy, intumescence coatings, wood protection, TGA-MS, TGA-IST16-GC-MS, DSC-microscopy

## Abstract

Fire-retardant intumescent coatings offer an effective means of enhancing the fire resistance of combustible substrates such as wood. These coatings have a complex chemical composition and, when exposed to temperatures above 200 °C, undergo an intumescent reaction accompanied by the release of non-flammable gases, forming an expanded, charred layer with low thermal conductivity. This provides thermal insulation and acts as a physical barrier against heat, oxygen, and flammable volatiles. In this study, the applicability of several thermoanalytical techniques for evaluating the performance of three different intumescent coatings applied to spruce wood was investigated. Simultaneous thermogravimetric analysis (TGA) and differential scanning calorimetry (DSC) showed that coating No. 3 was the most efficient, initiating substrate protection at the lowest temperature and reducing the combustion enthalpy by approximately 50% compared to uncoated wood. DSC-microscopy visualization enabled direct observation of the intumescent expansion, degradation of the carbonized protective layer, and delayed thermal decomposition of coated wood. Furthermore, a comparison between TGA-MS and TGA-IST16-GC-MS demonstrated the superiority of chromatographic separation for identifying evolved gaseous products. While TGA-MS is effective for detecting small gaseous species (e.g., H_2_O, CO_2_, formaldehyde), TGA-IST16-GC-MS enables the deconvolution of many degradation products evolving simultaneously, allowing for distinction between flame-retardant-related species, polymer backbone fragments, nitrogen-rich heterocycles, and small oxygenated molecules in the most effective coating.

## 1. Introduction

The use of fire-retardant coatings is one of the simplest, oldest, and most effective methods to protect substrates against fire. The concept of intumescence was first reported by Gay-Lussac in 1821 for the flame retardancy of textiles, while the term “intumescent” was first officially reported in an American patent in 1938 [1]. Earlier intumescent coatings had certain limitations, such as poor intumescent properties, inadequate paint qualities, and sensitivity to weathering [2]. Due to aesthetic requirements, improved durability, and better weather resistance, thin-film intumescent coatings are now produced as water-based, solvent-based, epoxy-based and hybrid coatings. Their composition, property comparisons, and commercial availability are discussed in a recent review article by Nazrun et al. [3]. Their use is increasing, as they are considered the most effective form of passive fire protection and have dominated this field since 2001 [2]. They are the preferred choice for architects and designers who wish to maintain the appearance of exposed surfaces.

The chemical composition of intumescent coatings is complex. They contain an acid source or catalyst that releases an inorganic acid when heated; an organic substance that forms a charred layer with the inorganic acid; an agent that releases large amounts of gases to cause the charred layer to expand; and a binder that holds all these components together and allows them to be applied to the surface. The addition of inorganic fillers, among which TiO_2_ is often used, has a synergistic effect on both the formation of the porous layer and the effectiveness of the resulting coating, as a ceramic-like structure is formed during the reaction [3,4,5,6]. The mechanism of the formation of the porous charred layer is described in detail in [3].

Intumescent coatings are primarily used to protect wood, structural steel, and tunnel walls from high temperatures and fire. These coatings, with typical thicknesses between 150 μm and 1500 μm, are indistinguishable from other coatings at room temperature. However, when exposed to temperatures typically between 200 °C and 250 °C, an intumescent reaction occurs, causing the coating to expand to 50 to 200 times its original thickness [5]. This results in a charred, porous, foam-like layer with low thermal conductivity. Consequently, the temperature of the material beneath the formed charred layer rises more slowly. In addition to the thermal effect, the intumescent coating also provides a mechanical barrier. The release of non-flammable gases into the environment during the intumescent reaction, along with the formation of the porous carbon layer, prevents flammable substances and oxygen from reaching the protected surface [7]. Intumescent coatings therefore extend the early stage of a fire, allowing for more time for safe evacuation. When applied to structural steel, which loses half its load-bearing capacity above 500 °C, the evacuation time is significantly increased compared to unprotected steel [8].

The coating itself has a temperature window of stability. The initial temperature marks the formation of a stable porous layer during the intumescent reaction of the coating at elevated temperature, while the final temperature indicates the onset of the breakdown of the formed porous carbon layer. Various thermoanalytical methods are powerful tools for understanding the mechanisms of the intumescent reaction and for determining the temperature window of stability. The unambiguous identification of volatile thermal decomposition products evolved during the intumescent reaction is possible through the simultaneous use of the coupled techniques Thermogravimetry-Mass Spectrometry (TGA-MS) and Thermogravimetry-Fourier-transform infrared spectroscopy (TGA-FTIR) [5]. Additionally, these techniques can be used in the development and optimization of intumescent materials. However, with TGA-MS coupling, interpretation of the mass spectra of gas mixtures containing large molecules is difficult due to their fragmentation [9,10]. In TGA-FTIR coupling, the measured infrared spectra are the sum of numerous individual spectra, making interpretation difficult even with the aid of a reference library [9,11]. In such cases, combining separation and identification techniques helps to unequivocally identify different evolved compounds [9,12]. The most used separation technique for gas mixtures is gas chromatography (GC). During the TGA analysis, gas samples can be taken at different degrees of thermal decomposition at predetermined temperatures. In a gas storage interface (IST), the gases are stored in numbered loops corresponding to specific sampling temperatures. The loops are analyzed off-line one after another using GC-MS. In GC, different molecules are separated according to their retention time, while each eluted component is determined separately with MS. Final identification of each separate component is based on a comparison of the measured *m*/*z* signals with a database [12,13,14]. Previous studies on intumescent coatings for wood predominantly rely on TGA and, less frequently, DSC, while coupled gas analysis techniques are not applied. In contrast, TGA-MS, TGA-GC-MS, and Py-GC-MS are well established for flame-retarded polymers and for untreated or impregnated wood pyrolysis, but not for coated wood systems [3,4,5,15,16,17,18].

This work addresses this gap by demonstrating the usefulness of advanced complementary thermoanalytical techniques for intumescent wood surface coatings, enabling comprehensive correlation between thermal degradation, volatile evolution, and char-forming behavior. We conducted a study on wood protection using intumescent coatings on spruce wood, as spruce is one of the most accessible types of wood in Slovenia and the most important commercial species, widely used in residential buildings (both for frame constructions and interiors) [19]. We sought to highlight the significant advantage of separating gas components by gas chromatography before MS analysis, compared to TGA-MS measurement alone. However, when many decomposition products evolve simultaneously, only the main products can be identified, and these may overlap with compounds present at low concentrations, which therefore cannot be detected. In-line TGA-GC-MS coupling systems are limited in the number of GC-MS measurements that can be processed during thermogravimetric analysis. Our focus is on off-line GC-MS analysis of gases released during TGA measurement and collected within a specific temperature range of interest in the interface storage oven. Once the last loop has been collected, the decomposition gases stored in the first loop are injected into the GC via a second heated transfer line by the GC carrier gas. Using this method, through TGA-IST16-GC-MS coupling, unambiguous determination of toxic gas products evolved is also possible. With Differential scanning calorimetry (DSC) analysis, it is possible to compare the enthalpy released during heating between bare wooden cubes and cubes with applied coatings, while coupled DSC-microscopy allows for visual observation of the formation of the intumescent charred layer.

## 2. Materials and Methods

Three different intumescent coatings, which were still in the development phase and intended for commercial use, were applied with a brush to Norway spruce wood cubes with dimensions of 5 × 5 × 4 mm^3^. The manufacturer has provided the following information: coatings No. 1 and No. 2 contain an acrylic binder. Coating No. 2 also contains TiO_2_, while coating No. 3 contains melamine resin (melamine formaldehyde). After drying under ambient conditions, simultaneous thermogravimetric analysis and differential scanning calorimetry (TGA-DSC) measurements were performed using a Mettler Toledo TGA/DSC1 instrument (Mettler Toledo, Greifensee, Switzerland) in 150 μL platinum crucibles (Mettler Toledo, Greifensee, Switzerland) under a high-purity air atmosphere (99.999%, Messer, Ruše, Slovenia) with a flow rate of 50 mL/min. The same type of crucible served as a reference. The initial isothermal programme for 10 min at 25 °C was followed by heating at a rate of 10 °C/min to 925 °C or 1000 °C. The masses of the intumescent coatings ranged from 5 mg to 7 mg, while the mass of the wooden cubes was approximately 35 mg. Curves obtained for coated wood cubes were compared with those for uncoated wood cubes. The baseline obtained under the same measurement conditions as the samples was subtracted each time.

For MS analysis of the released gas species, the TGA/DSC1 instrument was coupled with a Pfeiffer Vacuum ThermoStar GSD 320 T1 mass spectrometer (Busch Group, Maulburg, Germany) (TGA-MS). Evolved gases were introduced into the Mass Spectrometer through a 75 cm heated capillary at 180 °C.

In TGA-IST16-GC-MS analysis of the intumescent coating, the released gases can be stored in loops at 16 different analysis times, with the first loop used for purging [13,14]. The temperatures at which the gas species were collected were determined using a TGA curve obtained by conventional TGA-MS analysis, which shows the change in mass and intensity of MS signals as a function of temperature. We selected the temperatures at which the MS signals were highest, or the mass loss was greatest. In this analysis, we dried the sample to concentrate it in terms of solvent quantity and possibly reduce the intensity of the intumescent effect due to the limited size of the TGA furnace. The NIST mass spectrum library was used to identify the compounds on the total ion current (TIC) chromatogram. For the analysis, a Mettler Toledo TGA/DSC3+ instrument (Mettler Toledo, Greifensee, Switzerland) coupled with an Agilent Technologies 7890B GC gas chromatograph and an Agilent Technologies 5977B MSD mass spectrometer (both Agilent Technologies, Santa Clara, CA, USA) was used, with an IST16 from SRA Instruments (SRA Instruments Sas, Marcy l’Etoile, France) as the interface. The air flow rate in the TGA furnace was set to 30 mL/min and a 150 μL platinum crucible lined with a sapphire disc was used, with a sample mass of 5.2172 mg. The first gas was captured in the loop at the end of the isothermal programme, which ran for 10 min at 25 °C. This loop captures all impurities already present in the measuring system and is therefore intended for cleaning. This was followed by heating at a rate of 5 °C/min to 1000 °C. The IST transfer line and furnace were set to 240 °C. The temperature of the GC injector with sample division was set to 280 °C. The GC oven temperature programme was 10 min isothermal at 50 °C, followed by heating at a rate of 10 °C/min to 300 °C, and finally 15 min isothermal at 300 °C. An Agilent HP-5ms GC column with dimensions of 30 m × 250 μm × 0.25 μm was used. The carrier gas was helium with a flow rate in the column of 1.2 mL/min, provided under a controlled pressure of 67,569 Pa. In the GC injector, helium was split into a 3:1 ratio. The MS operated in full ion mode from 5 *m*/*z* to 550 *m*/*z*, using electron ionization (EI) at 70 eV and an electron multiplier voltage (EMV) gain factor of 1. The ion source temperature was set to 230 °C and the quadrupole to 150 °C.

For thermooptical measurements, a Mettler Toledo DSC1 instrument (Mettler Toledo, Greifensee, Switzerland) coupled with a Huber TC100 intracooler (Peter Huber Kältemaschinenbau SE, Offenburg, Germany) and equipped with an Olympus SC30 optical microscope (Olympus Soft Imaging Solutions GmbH, Münster, Germany) was used. The measurement of the intumescent coating itself was performed at a heating rate of 10 °C/min in an air atmosphere at a flow rate of 30 mL/min, using standard 40 µL gold crucibles (Mettler Toledo, Greifensee, Switzerland) in the temperature range from 25 °C to 700 °C. For the video recording of a wood cube coated with intumescent coating, the sample was heated from 30 °C to 600 °C at a rate of 10 °C/min in an air atmosphere at a flow rate of 30 mL/min using standard 40 µL aluminum crucibles (Mettler Toledo, Greifensee, Switzerland). The same type of crucibles used for the samples served as a reference. The Olympus Image Analysis Software analySIS 5.0 (Olympus Soft Imaging Solutions GmbH, Münster, Germany) was used to acquire, process, and measure images.

The graph analysis was performed using OriginPro 2017 software (OriginLab Corporation, Northampton, MA, USA).

Due to the significant changes in surface area and volume of the sample during the intumescent reaction, all samples were pre-tested in actual crucibles for thermal analysis by gradual heating and observation of expansion in a chamber furnace (Bosio, Celje, Slovenia) at intervals of 50 °C until reaching the desired final temperature or collapse of the sample due to combustion.

## 3. Results and Discussion

**Figure 1:**
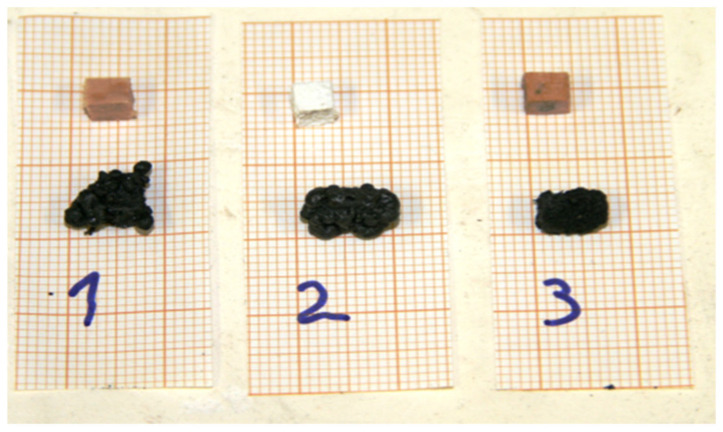
A photograph of spruce wood cubes measuring 5 × 5 × 4 mm^3^, protected with three different intumescent coatings, before thermal treatment (above) and as black charred samples after exposure to 400 °C (below). Coatings No. 1 and No. 3 were transparent after application, while coating No. 2 contained TiO_2_ and was therefore white.

After three different intumescent coatings were applied to separate spruce wood cubes, the samples were dried under ambient conditions. Coatings No. 1 and No. 3 were transparent after drying, while coating No. 2 was white due to its TiO_2_ content (Figure 1). When heated above a critical temperature, an intumescent material begins to swell and expand. This process produces a foamed, cellular charred surface layer that protects the underlying material from heat flux or flame. Visually, the swelling and expansion appear as black waves swollen at the surface of the material, and the final char exhibits a hemispherical shape with either a rough or smooth surface as shown in Figure 1.

### 3.1. TGA-MS and DSC Curves of Intumescent Coatings with DSC-Microscopy Visualization

**Figure 2:**
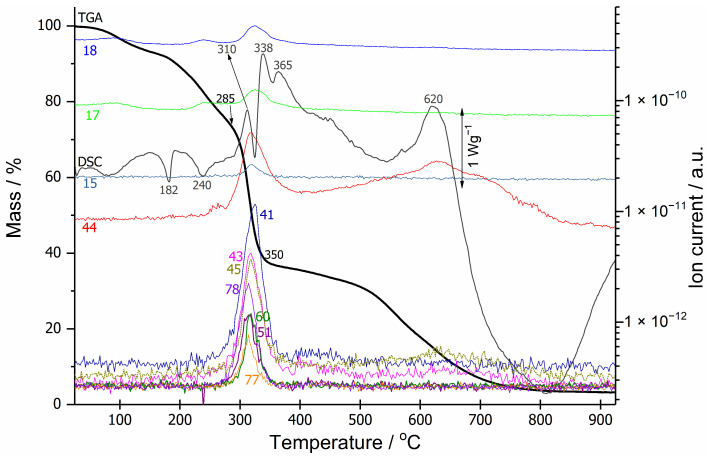
TGA-MS (bold black and colored lines) and DSC (dark grey line) curves of the intumescent coating No. 1.

**Figure 3:**
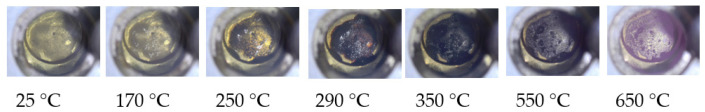
DSC-microscopy enables monitoring of the formation of a charred barrier during heating of intumescent coating and its breakdown.

From separate TGA-MS and DSC curves (Figure 2) and DSC-microscopy measurements (Figure 3) of coating No. 1 itself, the formation of a charred layer in the intumescent coating, when exposed to increased temperature, can be followed in detail. The endothermic peak at 182 °C, with an onset temperature of approximately 170 °C on the DSC curve, corresponds to melting of the binder. From approximately 240 °C to 350 °C, the intumescent reaction occurs, resulting in the evolution of inert gaseous species: water (*m*/*z* = 18, 17) and CO_2_ (*m*/*z* = 44, 45, 43). Acetic acid was identified by mass peaks at *m*/*z* = 43 (CH_3_CO^+^), *m*/*z* = 45 (COOH^+^), *m*/*z* = 60 (CH_3_COOH^+^) and *m*/*z* = 15 (CH_3_^+^). Fragments characteristic of aromatic rings, *m*/*z* = 50 (C_4_H_2_^+^) and *m*/*z* = 51 (C_4_H_3_^+^) were also detected, while *m*/*z* = 41 (C_3_H_5_^+^) and *m*/*z* = 42 (C_3_H_6_^+^) are more indicative of aliphatic or olefinic fragments, which could originate from alkyl side-chains on aromatic systems (for clarity, only 51 and 41 are presented in Figure 2). Peaks at *m*/*z* = 78 (C_6_H_6_^+^) and *m*/*z* = 77 (C_6_H_5_^+^) also appeared, indicating the release of benzene [20]. Combustion of the carbonaceous layer at temperatures above 500 °C is clearly seen on the TGA-MS curve, where only the evolution of CO_2_ (*m*/*z* = 44, 45, 43) is observed, as well as in the DSC-microscopy snapshots (Figure 3). The coating burned completely, leaving around 3% residue at a final temperature of 925 °C.

The complete thermal response of intumescent coating No. 1, the formation and collapse of the charred layer, is recorded on video and available as Supplementary File (Video S1).

**Figure 4:**
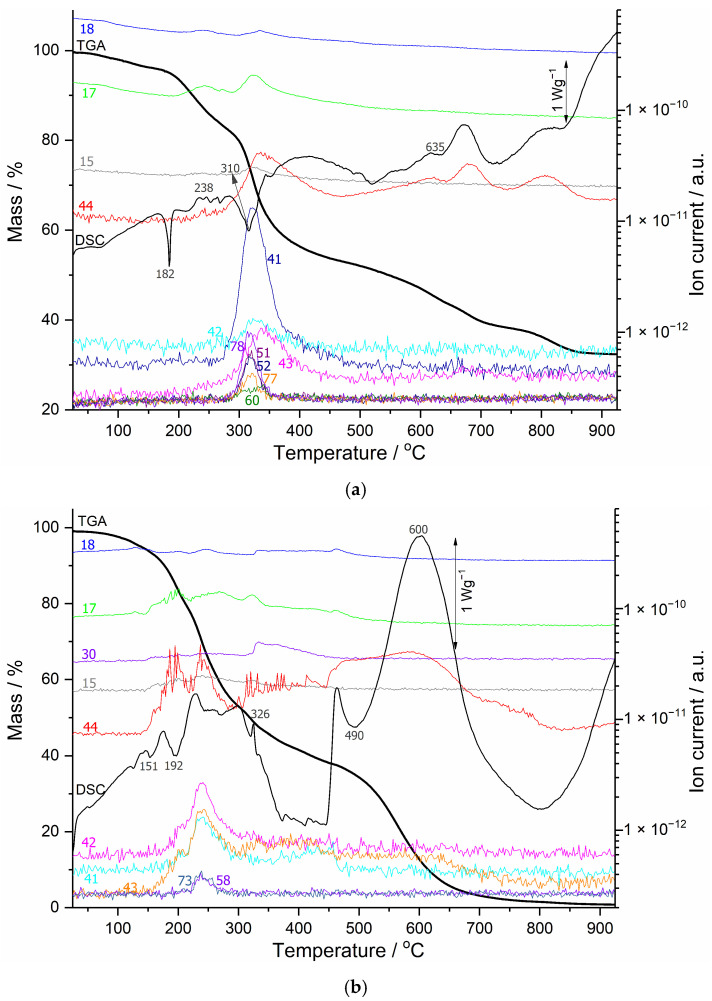
(**a**) TGA-MS (bold black and colored lines) and DSC (dark grey line) curves of the intumescent coating No. 2; (**b**) TGA-MS (bold black and colored lines) and DSC (dark grey line) curves of the intumescent coating No. 3.

Using TGA-MS and simultaneous DSC signals, we also examined coatings No. 2 and No. 3, as shown in Figure 4a,b. In coating No. 2, same as for coating number No. 1, the DSC curve displays a narrow endothermic peak at 182 °C, which we attribute to the melting point of the binder, followed by an intumescent reaction in the temperature range from 238 °C to 465 °C, with the release of CO_2_ and H_2_O gases resulting from the combustion of organic compounds and fragmentation. This indicates the presence of acetic acid, benzene, and fragmentation characteristic of the evolution of aromatic rings and aliphatic or olefinic fragments. At temperatures above 550 °C, the exothermic peaks on the DSC curve, together with an MS signal of *m*/*z* = 44 (CO_2_), indicate the combustion of char; so, no water is detected in this temperature range. The combustion reaction ends at approximately 850 °C, and the remaining mass on the TGA curve stabilizes at the highest value of all coatings, at 32%. The residue in the crucible is a white powder, indicating the presence of inert TiO_2_.

For coating No. 3, the DSC curve shows the onset of an intumescent reaction with several endothermic peaks already appearing in the range of 150 °C. The evolution of characteristic fragments for water, carbon dioxide, and organic molecules is observed up to 490 °C, when the final 30% of the charred sample mass begins to burn, releasing CO_2_. The mass loss is complete by 925 °C. In this MS measurement, the mass peak at *m*/*z* = 30 was also observed, which could correspond to formaldehyde (CH_2_O^+^).

### 3.2. TGA and DSC Curves Obtained for Coated Wood Cubes Compared with Uncoated Wood

**Figure 5:**
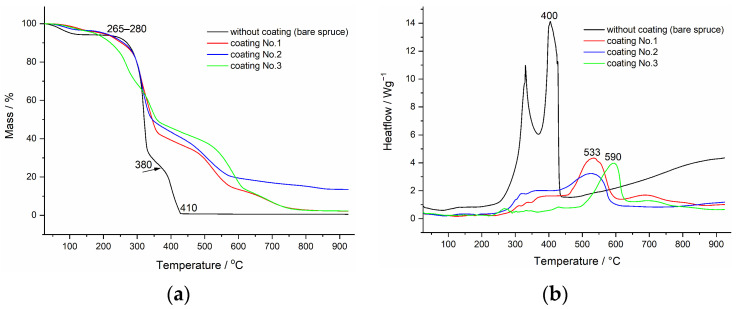
(**a**) TGA curves obtained for coated wood cubes (colored lines) compared with uncoated wood (black line); (**b**) DSC curves obtained for coated wood cubes (colored lines) compared with uncoated wood (black line).

From simultaneous TGA (Figure 5a) and DSC measurements (Figure 5b), the effectiveness of the separate intumescent coatings can be easily compared using only four measurements. After the initial mass loss due to water content, at temperatures above 250 °C, unprotected spruce wood (black line) begins to undergo thermal decomposition. In the first step hemicelluloses and non-crystalline cellulose fibers degrade, while in the second successive step, from 380 °C onwards up to the final 410 °C, thermal decomposition of crystalline cellulose occurs. Both steps of thermal decomposition are clearly visible from the distinct exothermic effects on the DSC curve (Figure 5b). Lignin decomposes throughout the entire temperature range [21,22].

For protected (coated) samples, wood decomposition begins at the same temperature as for unprotected sample, except for coating No. 3 (green line), where thermal decomposition starts at approximately 20 °C lower temperature. This is due to the earlier intumescent reaction of the coating. As shown by comparing the coating alone (Figure 2 and Figure 4) and this figure, the formation of a charred barrier also starts above 200 °C; so, the mass loss in the TGA curves (Figure 5a) results from simultaneous reactions in the coating and the thermal decomposition of the wood.

At around 350 °C, a porous charred layer is formed; and consequently, a decrease in the rate of mass loss is observed on the TGA curves of protected wood compared to unprotected wood (Figure 5a). The mass of the coated wood samples then decreases at a moderate rate up to approximately 500 °C, where the charred layer begins to combust, becomes progressively thinner, and is no longer able to provide thermal and mechanical protection to the substrate.

From the corresponding DSC curves (Figure 5b), the enthalpy of the exothermic reaction is much lower for protected samples (Table 1). The final exothermic peak with the highest intensity corresponds to the combustion of the charred layer together with the wood residue beneath. This strongly confirms that intumescent coatings mainly prolong the early stage of fire development. The most probable reason is the dilution of the pyrolysis gases from the wood with non-flammable gases, which are formed during the intumescent reaction (water, CO_2_) and the formation of an oxygen barrier.

Of the samples tested, coating No. 3 exhibits the best properties: it began to protect the substrate at the lowest temperature, resulting in slightly lower mass loss compared to the other two. More importantly, the enthalpy of the combustion reaction is halved compared to the unprotected sample.

**Figure 6:**
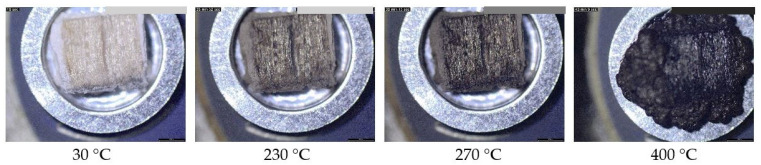
Digital images of a halved, coated wood cube on DSC1 were recorded using an optical microscope. Selected images are shown at temperatures typical for the development of the intumescent process. The side with intumescent coating no. 2 was on the underside of the wood and is also visible as a thin layer on the side. The upper side of the wood was unprotected due to the splitting of the cube, allowing for observation of the degradation of the wood itself.

Using digital image capture, we also recorded a video of a halved coated wood cube on DSC1 with an optical microscope (Figure 6, Video S2). The side with intumescent coating No. 2 was on the underside of the wood and is visible as a thin layer on the side. The upper side of the wood was unprotected due to the splitting of the cube, allowing for observation of the degradation of the wood itself. The gradually darkening color of the bare wood, which becomes more noticeable at temperatures above 200 °C, results from the formation of oxidized decomposition products such as quinones. The video recording is included in the Supplementary File (Video S2). To determine the temperature in °C in this video, multiply the time shown in minutes on the recording by approximately 10.

### 3.3. TGA-IST16-GC-MS Analysis of Dried Intumescent Coatings No. 3

Based on the TGA-MS measurements (Figure 2 and Figure 4), the results indicate the presence of small gas molecules (water, carbon dioxide, formaldehyde), as interpreted in the previous section. However, extensive fragmentation of organic compounds prevents reliable interpretation, especially if the content of the coating components is largely unknown.

On the TGA-MS graph of dried intumescent coating No. 3 (Figure S1), an almost real situation after a TGA-MS measurement can be observed. The large number of curves showing peaks can be grouped by temperature range, by molecules predicted to vaporize, and matched to the TGA curve.

Chromatographic separation is therefore necessary to identify the simultaneously evolved products, which means coupling a TGA instrument to a GC-MS system. In our coupling system, the TGA decomposition products are transferred to the heated IST storage oven through a heated transfer line at 250 °C, where up to 16 gas samples are stored in 250 μL loops. In Figure S1, vertical lines indicate the selected temperature positions at which gas is captured into the loops. In regions of greater mass loss, the number of loops is increased to obtain more accurate information. The temperature programme for the 15 selected gas capture areas into IST16 is presented in Table 2. Subsequently, the gas samples are automatically and sequentially analyzed by GC-MS; volatile products with masses up to *m*/*z* = 250 can be detected. Summing the intensity of all the ions yields the total ion chromatogram (TIC), which indicates the amount of gas leaving the GC column at any given time. Due to the intense intumescent reaction observed in both videos (Videos S1 and S2), only a small sample mass of 5.2 mg was analyzed. This sample had been previously dried to increase the concentration of the components.

A complex intumescent coating produces several decomposition volatiles, with up to 30 compounds detected and identified in a single loop. Table 3 lists the identified compounds mentioned in the following text at their retention times, Table S1 lists integrated peak areas for identified compounds in each loop and are traceable via retention time.

**Figure 7:**
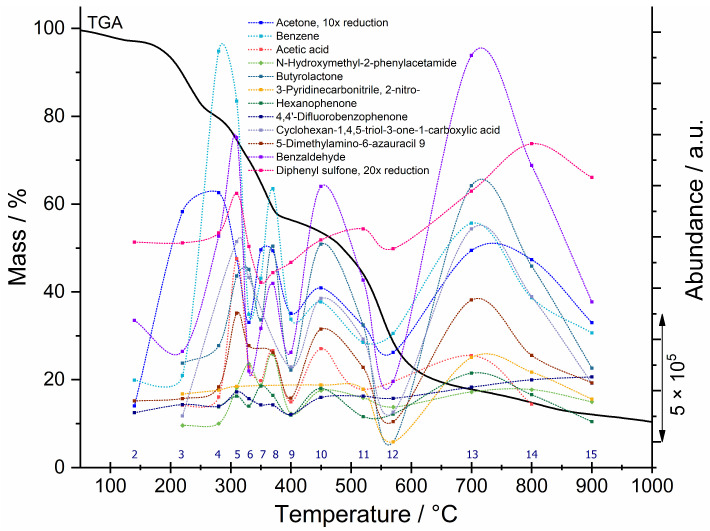
Results of TGA-IST16-GC-MS analysis of intumescent coatings No. 3. TGA curve (bold black line) and fitted curves representing components in loops detected through GC-MS analysis (colored lines).

In Figure 7, the results of TGA-IST16-GC-MS analysis of intumescent coating No. 3 are shown. The results for selected gas components, based on GC-MS analysis for 14 loops (2–15), are added to the TGA curve (bold black line). As previously mentioned, the first loop is used for cleaning. A series of discrete data points representing components in loops are connected into fitted curves (colored lines) using a parametric equation for a modified Bezier curve available in OriginPro 2017 software.

GC-MS analysis confirmed that intumescent coatings are complex, multi-component formulation systems, which are difficult to interpret. However, we attempted to classify the detected combustion products into groups as follows:

(i) Products that indicate flame-retardant (FR) chemistry. These compounds all appear in the TIC at higher retention times. Aromatic sulfone, specifically diphenyl sulfone (*t*r = 26.903 min), indicates the presence of sulfone-based FR additives [23], formed by the scission of sulfone bridges during thermal degradation in sulfone-based polymers such as polysulfone, poly(ether sulfone)). 4,4′-difluorobenzophenone (*t*r = 23.200 min) was also detected, which may indirectly indicate the presence of fluorinated aromatic FR polymers and benzophenone-based FR frameworks, where it is used as a monomer for the synthesis of polyether ether ketone (PEEK) thermoplastic polymers [24]. Diphenyl ether (*t*r = 20.829 min) may indicate polymer backbone cleavage, such as in PEEK, or may be a precursor or breakdown product of brominated or non-halogenated FR systems. At the same retention time, at temperatures between 370–400 °C, barbituric acid derivatives, as nitrogen-rich heterocycles, were more likely detected [23].

(ii) Combustion products of aromatic FR polymers. Benzene, toluene, o-xylene and p-xylene, naphthalene, o-hydroxybiphenyl, hexanophenone, and benzaldehyde were detected. The concurrent detection of benzene, toluene, and (o-, p-)xylene is consistent with high-temperature (in loops between 310 °C and 370 °C) methyl transfer and disproportionation reactions among alkylated aromatics during polymer pyrolysis, written as stoichiometric equation 2 C_7_H_8_ → C_6_H_6_ + C_8_H_10_ (toluene → benzene + xylene) [23,25]. Benzaldehyde was detected as an aromatic carbonyl compound and is interpreted as a secondary oxidation product formed during thermal degradation of aromatic compounds at high temperature, rather than as an intentionally added flavor or fragrance component [20].

(iii) Nitrogen-containing species. These strongly suggest nitrogen-based flame retardancy and thus support char formation and gas-phase flame inhibition: ethanediamide, amino alcohols, imidazoles, pyrazoles, oxazolidinones, proline derivatives [18,26].

(iv) Oxygenated small molecules, which are products of high-temperature polymer breakdown: acetone, methacrolein, butyrolactone, hydroxy acids, aldehydes and ketones [18,23].

Figure 7 clearly shows that in the temperature range between 500 °C and 600 °C, the content of gas components decreases (loops 11 and 12). This is explained by the increased concentration of the light gas component CO_2_ in the total gas mixture in a loop, as the carbonized protective degradable barrier begins to combust. All light gas components are part of the argon carrier gas, which produces an intense chromatographic peak in the retention time range of 1.142 to 1.391 min. Carbon monoxide (CO) and nitrogen (N_2_) gas molecules were also detected in several loops within this time range.

From the TGA-IST16-GC-MS analysis of coating No. 3, only the detection of nitrogen-rich heterocycles (e.g., barbituric acid derivatives) provides supporting evidence for melamine/triazine-based components.

## 4. Conclusions

The aim of our work was to demonstrate the usefulness of various thermoanalytical techniques for studying intumescent coatings, particularly for wood surface applications. We highlighted several approaches.

The TGA and DSC analysis results show that Coating No. 3 provides the most effective fire protection, as its intumescent reaction occurs 20 °C earlier than the thermal decomposition of uncoated spruce wood. This early formation of a porous char layer reduces mass loss, slows the thermal degradation of the wood, and limits heat and oxygen transfer. DSC analysis demonstrates the superior performance of Coating No. 3, showing a reduction of about 50% in combustion enthalpy compared to unprotected wood. This confirms the effective dilution of flammable pyrolysis gases by non-flammable gases (such as water vapor and CO_2_) released during intumescence, along with the formation of an oxygen-insulating char layer, thereby enhancing fire protection performance.

Using visualization with DSC-microscopy, we effectively presented and understood the progression of the intumescent reaction and the degradation of the carbonized protective barrier in the coating, as well as the delay in thermal degradation of coating-protected wood compared to bare wood.

This study clearly demonstrates the unique advantage of the TGA-IST16-GC-MS system over conventional TGA-MS. While TGA-MS is effective for detecting small gaseous species (e.g., H_2_O, CO_2_, formaldehyde), extensive fragmentation severely limits chemical interpretation for complex commercial coatings. In contrast, TGA-IST16-GC-MS enables time-resolved gas capture, chromatographic separation, and compound-level identification, allowing for deconvolution of many degradation products evolving simultaneously. This capability makes it possible to distinguish flame-retardant-related species, polymer backbone fragments, nitrogen-rich heterocycles, and small oxygenated molecules, which cannot be reliably resolved by TGA-MS alone. Temperature-resolved tracking of individual molecules enables the linking of specific compounds to distinct degradation stages. This approach is particularly powerful for intumescent coatings, where multiple reactions—binder melting, intumescence, char formation, additive decomposition, and char combustion—occur simultaneously.

The combined use of TGA-DSC, TGA-MS, thermoptometry (DSC-microscopy), and TGA-IST16-GC-MS represents a powerful, complementary multi-technique framework that is particularly well suited for the rapid and mechanistically informed evaluation of intumescent coatings for wood. As all methods require small sample quantities, short analysis times, and minimal sample preparation, the framework enables early identification of high-performance formulations, guides formulation optimization, and provides strong predictive insight into fire behavior, making it a highly valuable tool for both research and industrial development of fire-protective coatings for wood.

## Figures and Tables

**Table 1:** Comparison of the enthalpy values of combustion reaction for protected and bare wood samples determined by differential scanning calorimetry (DSC).

**Coating**	**Enthalpy** **/** **J/g**	**Reduction in Released Heat** **/%**
Bare wood	5860	
Coating No. 1	4079	30.4
Coating No. 2	3059	47.8
Coating No. 3	2828	51.7

**Table 2:** Temperature program for capturing gas products in IST16 loops.

**Loop Number**	**1**	**2**	**3**	**4**	**5**	**6**	**7**	**8**	**9**	**10**	**11**	**12**	**13**	**14**	**15**
Sample collection temperature/°C	50	140	220	280	310	330	350	370	400	450	520	570	700	800	900

**Table 3:** Identified compounds and their retention times from TGA-IST16-GC-MS analysis.

**Composition Name**	**Retention Time/min**
Acetone, Butane; 3-Amino-2-oxazolidinone 9; Pentane, 1-(2-propenyloxy)-	1.535
D-Proline; Methacrolein;	1.717
Benzene	2.163
5-Dimethylamino-6-azauracil 9; AS-Triazine-3,5(2H,4H)-dione, 6-(dimethylamino)-	2.354
Acetic acid	2.433
Ethanediamide; Butanoic acid, 3-amino-	2.562
Toluene; N-Hydroxymethyl-2-phenylacetamide	3.332
2,4,7-Octanetrione; 2-Hexanone, 4-hydroxy-3-propyl-	3.784
1H-Imidazole, 4,5-dimethyl-; 1H-Imidazole-4-carboxaldehyde	4.891
o-Xylene	5.763–5.777
p-Xylene	6.070
Butyrolactone	8.339–8.403
Benzaldehyde	11.163–11.186
3-Pyridinecarbonitrile, 2-nitro-	12.327
2-Furanmethanol, tetrahydro-5-methyl-, trans-; 2H-Pyran-2-ol, tetrahydro	14.670
Hexanophenone	14.914
3-Morpholinone, 5-methyl-6-phenyl-; 2H-Pyran-2-ol, tetrahydro-;	16.882
Naphthalene; 1-[2-Pyridyl]-2,2-dimethyl-2-morpholino ethanol	17.379
o-Hydroxybiphenyl; Barbituric acid; 5,5-dipropyl-;2-biphenyl ester; Diphenyl ether	20.829
4,4′-Difluorobenzophenone	23.200
Diphenyl sulfone	26.903

## Data Availability

The original contributions presented in this study are included in the article/Supplementary Material. Further inquiries can be directed to the corresponding author.

## Supplementary Materials

The following supporting information can be downloaded at: https://www.mdpi.com/article/10.3390/polym18020202/s1, Figure S1: TGA-MS of dried intumescent coating No. 3 with loops setup; Table S1: Integrated peak areas for identified compounds in each loop, traceable via retention time; the videos are available via https://doi.org/10.5281/zenodo.18033853, Video S1: The complete thermal response of intumescent coating No. 1, the formation and collapse of the charred layer; Video S2: The formation of a charred barrier on a halved, coated wood cube.
