# Supplementary material for: Complementary Techniques of Thermal Analysis as a Tool for Studying the Properties and Effectiveness of Intumescent Coatings Deposited on Wood
Source: Polymers (Basel). 2026 Jan 12;18(2):202. doi: 10.3390/polym18020202 (PMC12845888; doi:10.3390/polym18020202)
Supplement: Supplementary file 1 [file polymers-18-00202-s001.zip › Table S1. Integrated peak areas for identified compounds in each loop, traceable via retention time.pdf]

Table S1. Integrated peak areas for identified compounds in each loop, traceable via retention time.

**Loop number**	**2**	**3**	**4**	**5**	**6**	**7**	**8**	**9**	**10**	**11**	**12**	**13**	**14**	**15**
Sample collection temperature / °C	140	220	280	310	330	350	370	400	450	520	570	700	800	900
Retention time / min	Peak area / a.u.													
1.535	1404025	8984348	9732375	7136139	4665825	7491038	7466933	5019791	6011700	4555773	3493626	7474665	7114881	4659527
1.717		101452		781346	642217			292331	559973	400576	115980	831328	567167	228022
2.163	241455	259107	1525966	1330068	498017	639375	988552	478891	546503	388774	423680	853535	562823	425234
2.354	159998	169053	214462	502377	375256		349355	170685	439776	290727	78703	553687	337735	230140
2.433		143526	175204	715723	292258	238873	356456	155750	363406	205327		337368	147233	
2.562			117512	155505	103505			131360					65300	
3.332		64440	72207	212106	305063	220966	342799	109769	200899	172432	135873	195022	204550	156504
3.784				469334	227351		202067		317106	221507		426415		
3.982				368241	121523				235560			122115		
4.891				158357	171678	148628	105053		171519			150929	154894	150014
5.763-5.777					159831	339871	76603							
6.070				219733	625701	194264								
8.339-8.403		307279	375656	647469	673079	476065	764452	279844	771494	456610		1000624	686397	287815
11.163-11.186	473768	352980	803182	1187427	277514	443245	619157	349932	997863	631987	236482	1508916	1079043	546678
12.327		186910	202151	216709					222041	205717		329506	272561	167410
14.670		123363	165295		227014	64778	269222			127042				125652
14.914			136381	178380	139858	218296	181325	104818	208663	98735		268231	184037	78888
16.882		75687	61193	139843	70742		83692		128392	49384		277872	129970	35608
17.379				68858	45409		122848		161132					
20.829	65940	84258	72104	101969	82435	27398	119371	146707	165400	143350	79393	173379	191555	156093
23.200	114017	146011	139231	194979	168999	144341	144174	107222	173152	178606	170205	213274	242417	253809
26.903	15593093	15538896	16322732	19393644	15257607	12457497	13205926	14013005	15765532	16638722	15092181	19573564	23279684	20647463
