# Supplementary figures and images for: Complementary Techniques of Thermal Analysis as a Tool for Studying the Properties and Effectiveness of Intumescent Coatings Deposited on Wood
Source: Polymers (Basel). 2026 Jan 12;18(2):202. doi: 10.3390/polym18020202 (PMC12845888; doi:10.3390/polym18020202)

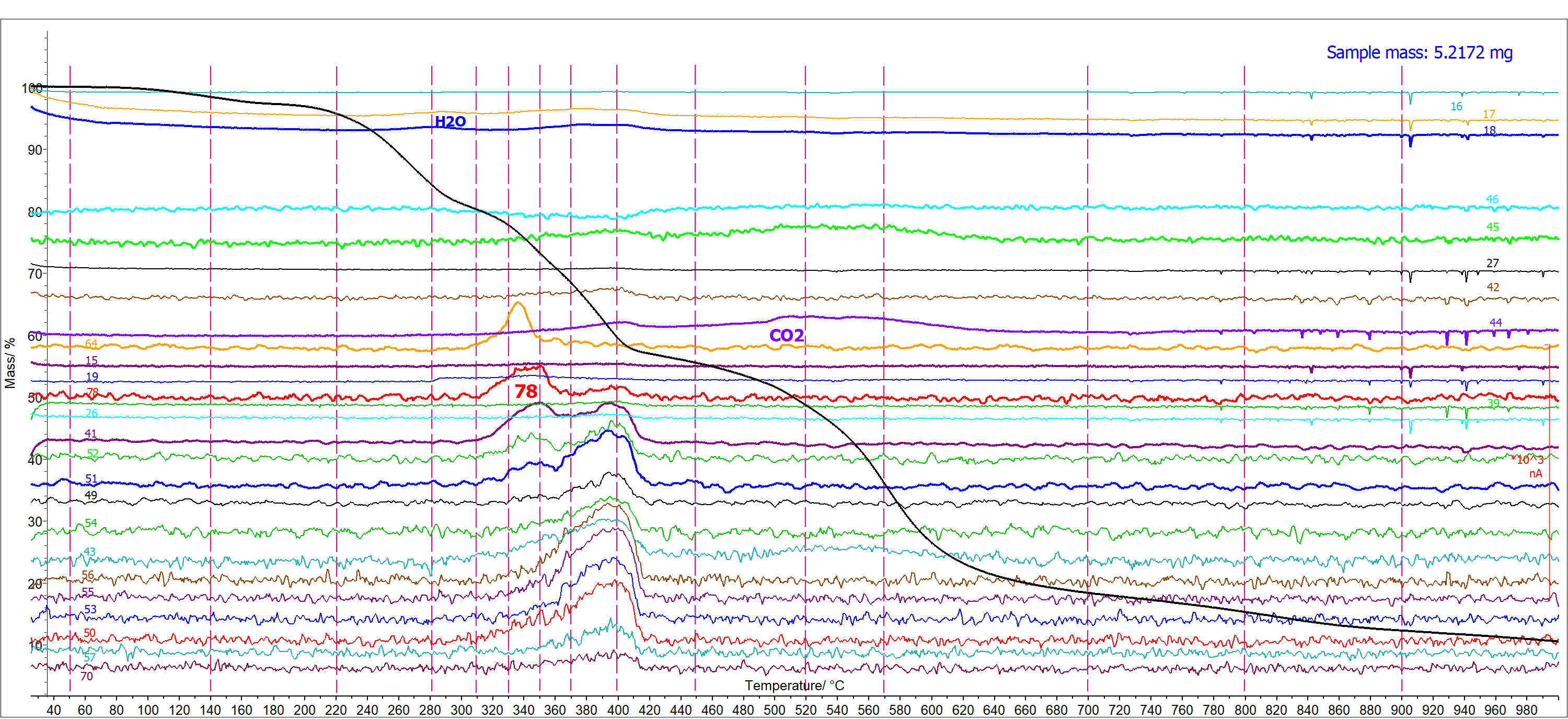

Supplement: Supplementary file 1 [file polymers-18-00202-s001.zip › Figure S1 TGA_MS of dried intumescent coating No 3 with loops setup.tif]
